# Supplementary material for: Physics‐Informed Inverse Design of Programmable Metasurfaces
Source: Adv Sci (Weinh). 2024 Sep 5;11(41):2406878. doi: 10.1002/advs.202406878 (PMC11538652; doi:10.1002/advs.202406878)
Supplement: Supplementary file 1 — Supporting Information [file ADVS-11-2406878-s001.docx]

Supporting Information

Physics-informed Inverse Design of Programmable Metasurfaces

*Yucheng Xu, Jia-Qi Yang, Kebin Fan,*^*^ *Sheng Wang, Jingbo Wu, Caihong Zhang, De-Chuan Zhan, Willie J. Padilla,* *and Biaobing Jin,*^*^ *Jian Chen, Peiheng Wu*

Contents

[1. Device fabrication Process 3](#_Toc173706318)

[2. Schematic diagram and experiment photograph of the beam steering measurements 3](#_Toc173706319)

[3. Detailed discussion about the difficulty in achieving phase tuning over 270° 4](#_Toc173706320)

[4. Modified coupled mode theory 8](#_Toc173706321)

[5. Evaluation and discussion of intersection points with maximum phase difference 11](#_Toc173706322)

[6. Inverse design procedure 12](#_Toc173706323)

[7. Detailed explanation of forward and backward loss functions in ResMLP network 13](#_Toc173706324)

[8. Inversely designed reconfigurable metasurface by PIID 16](#_Toc173706325)

[9. Physics learned from the ResMLP network 17](#_Toc173706326)

[10. The relationship of geometry and mode parameters 18](#_Toc173706327)

[11. Multiple encoding schemes (2-bit, 1-bit and ‘tri-states’) for THz beam steering 19](#_Toc173706328)

[12. Asymmetric beam deflection energy distribution of the device with 2-bit programmable capability 22](#_Toc173706329)

[13. Measurements of high-order diffractions of the programmable metasurfaces 22](#_Toc173706330)

[14. The simulation results of the beam steering performance 23](#_Toc173706331)

[15. Performance comparison of various devices in Terahertz beamsteering 25](#_Toc173706332)

[References 26](#_Toc173706333)

1. Device fabrication Process

Detailed process of the sample fabrication is shown in Figure S1.


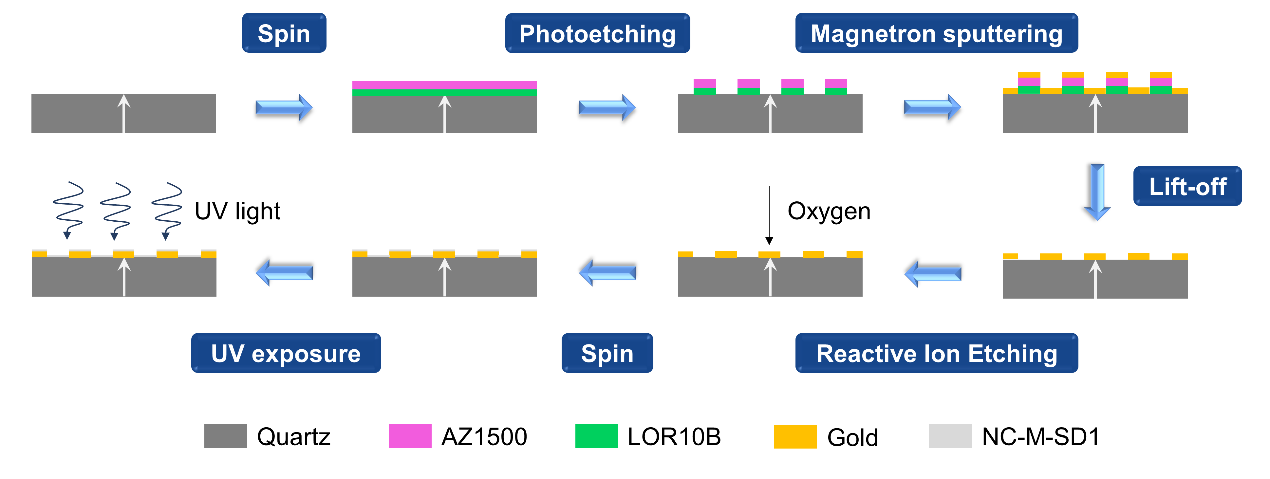


Figure S1. Detailed flow chart of the sample fabrication process. Both the top and bottom substrates undergo the same microstructure fabrication process.


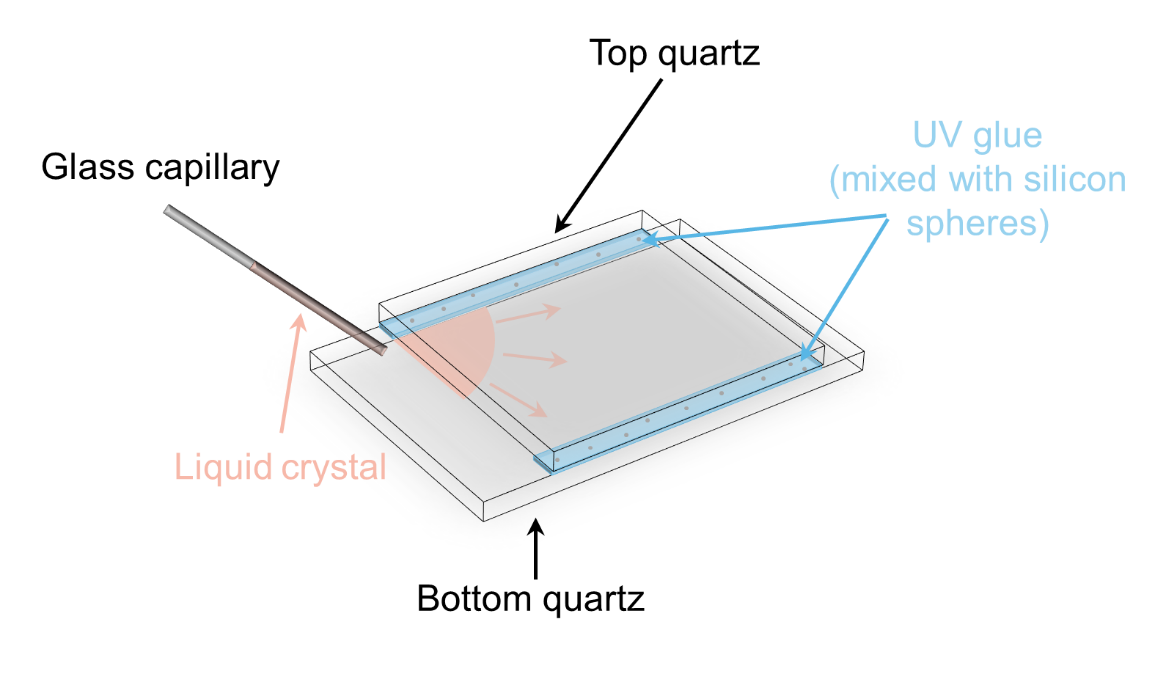


Figure S2. Illustration of the packaging of the LC box and the injection of LC

1. Schematic diagram and experiment photograph of the beam steering measurements

The optical fiber THz time-domain spectroscopy system (THz photonics TPF15K) is adopted for measuring THz beam deflection. Due to limited geometry size of the THz beam emitter and receiver, the minimum suitable incident angle is 10.5°. The scanning angle range of the receiver is from 10° to 90°. Rotation of the receiver relies on the rotation stage. Figure S3 shows the schematic and photograph in actual experiments of the measurements.


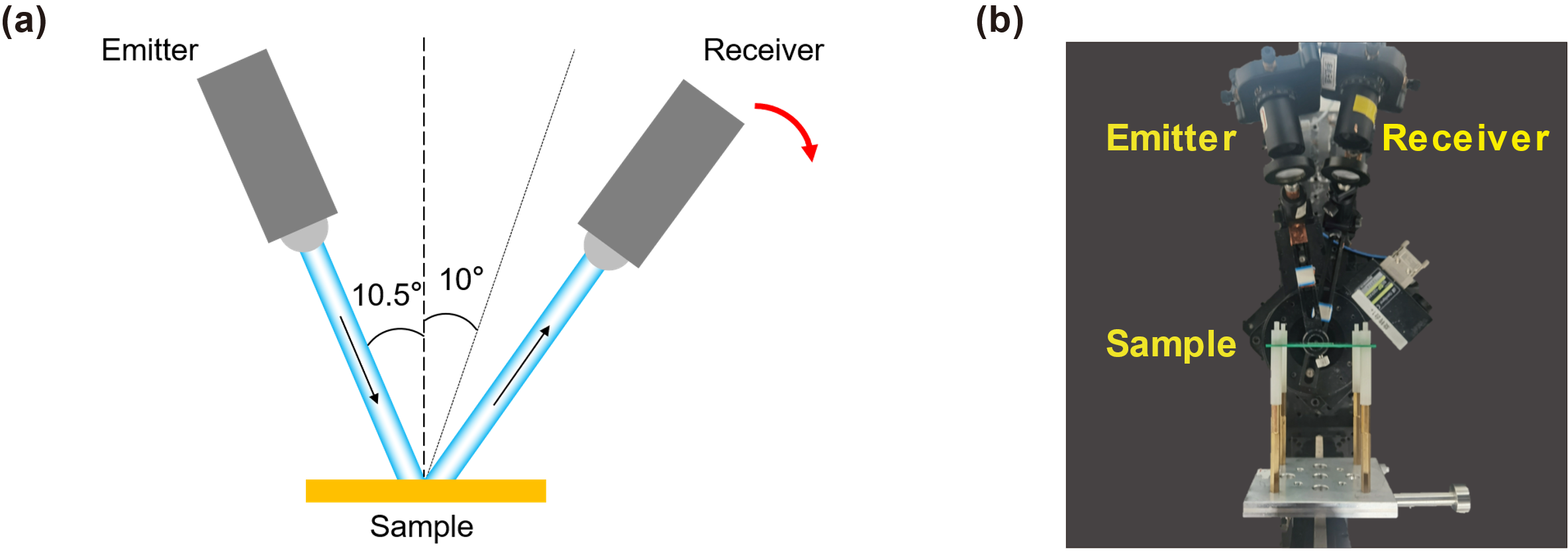


Figure S3. Measurement system of beam steering performance (a) schematic diagram (b) photograph of the experimental setup.

1. Detailed discussion about the difficulty in achieving phase tuning over 270°

In this section, we will demonstrate that achieving phase tuning over a 270-degree range while maintaining a high reflection amplitude necessitates a carefully designed large radiative loss rate for metasurfaces. This design becomes particularly challenging when the materials in the metasurfaces are lossy.

For a simple reflective metasurface modulator with a metal-insulator-metal configuration, where the spacing is filled with liquid crystal, the reflection can be described using coupled mode theory as [3]

$$\begin{aligned} r_{m}\left( \omega\right)=-1+\frac{2\gamma}{j\left( \omega-\omega_{0} \right)+\left( \delta+\gamma\right)}. \#\left( S1 \right) \end{aligned}$$

where $\omega_{0}$, $\gamma$ and $\delta$ are the mode frequency, radiative loss rate, and the intrinsic material loss rate. The complex reflection coefficient can be separated into its real and imaginary parts as $r_{m}(\omega)=r_{re}(\omega)+jr_{im}(\omega)$. Both parts follow the constraint of

$$\begin{aligned} \left( r_{re}\left( \omega\right)+\frac{\delta}{\gamma+\delta} \right)^{2}+r_{im}\left( \omega\right)^{2}=\left( \frac{\gamma}{\gamma+\delta} \right)^{2}\#\left( S2 \right) \end{aligned}$$

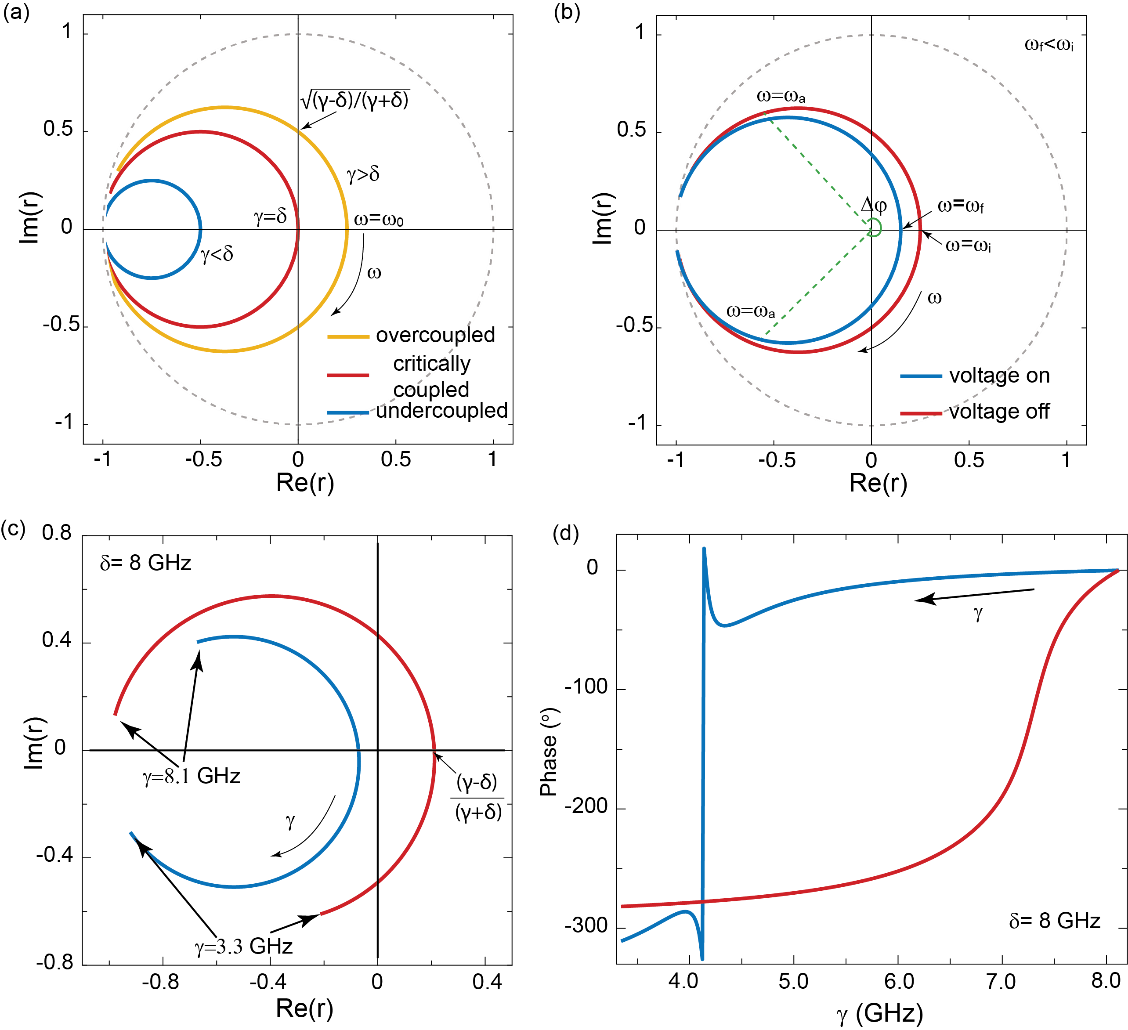


Figure S4. (a) Trajectories of complex reflection coefficients for three coupling conditions: undercoupled state ($\gamma<\delta$); critically coupled state ($\gamma=\delta$); and overcoupled state ($\gamma>\delta$). (b) Trajectories of the complex reflection coefficients of an LC based tunable metasurface before and after switching. (c) The trajectories of reflections with the radiative loss rate varying from 8.1 GHz to 3.3 GHz at operating frequencies of $\omega_{a}=0.41$ THz (blue curve) and 0.465 THz (red curve), respectively. The resonant frequency is assumed to redshift from 0.46 THz to 0.4 THz simultaneously. The material loss rate is approximated as a constant with $\delta=3.3$ GHz. (d) The corresponding phase change a function of the radiative loss rate at operating frequencies of $\omega_{a}=0.41$ THz (blue curve) and 0.465 THz (red curve), respectively.

which indicates that the real and imaginary parts of the reflection coefficient are on a circle centered at $\left( -\frac{\delta}{\gamma+\delta}, 0 \right)$ with a radius of $\frac{\gamma}{\gamma+\delta}$, as shown in Figure S4. When the metasurface is operated at the undercoupled state, the resonant system is highly dissipative. As a result, the total phase change in the monitored frequency range will be smaller than 180 degrees since the trajectory of the reflection coefficient only lies in the 2^nd^ and 3^rd^ quadrants. When the trajectory crosses over the real axis, the metasurface is operating at the resonant frequency. As the radiative loss rate is balanced by the material loss rate, the metasurface achieves perfect absorption at the resonant frequency. A further increase of the radiative loss rate leads to a less lossy metasurface operating at overcoupled state. As a result, the total phase change across the frequency range can be larger than 180 degrees. Then to achieve large phase tuning after tuning, it requires at least one of the operating states for metasurface is overcoupled.

Next, we will analyze the dependency of the maximal phase change on the resonator properties. When there is no frequency shift for the resonant frequency after tuning, the maximum phase change at some fixed frequency will be 180 degrees when the metasurface operates from the overcoupled state to the undercoupled state, and vice versa. However, when the resonant frequency is shifting during the modulation, the analysis will be complex. For example, the applied bias on the liquid crystal will shift the resonance from initial resonant frequency of $\omega_{i}$ to lower frequency of $\omega_{f}$($\omega_{i}>\omega_{f}$) due to the increased refractive index of the LC. Furthermore, according to our calculation, the radiative loss rate $\gamma_{i}$ will be changed to $\gamma_{f}$ and the material loss rate nearly remains the same due to the loss tangents of all materials change negligibly. The complex reflection coefficients before and after modulation can be further derived as

$$\begin{aligned} r_{mi}\left( \omega\right)=\frac{-j\left( \omega-\omega_{i} \right)+\left( \gamma_{i}-\delta\right)}{j\left( \omega-\omega_{i} \right)+\left( \gamma_{i}+\delta\right)}=\frac{-\Delta\omega_{i}^{2}+\left( \gamma_{i}^{2}-\delta^{2} \right)-j2\gamma_{i}{\Delta\omega}_{i}}{\Delta\omega_{i}^{2}+\left( \gamma_{i}+\delta\right)^{2}}\#\left( S3 \right) \end{aligned}$$

where $\Delta\omega_{i}=\omega-\omega_{i}$. After tuning, the reflection coefficient will be given as

$$\begin{aligned} r_{mf}\left( \omega\right)=\frac{-j\left( \omega-\omega_{f} \right)+\left( \gamma_{f}-\delta\right)}{j\left( \omega-\omega_{f} \right)+\left( \gamma_{f}+\delta\right)}=\frac{-\Delta\omega_{f}^{2}+\left( \gamma_{f}^{2}-\delta^{2} \right)-j2\gamma_{f}{\Delta\omega}_{f}}{\Delta\omega_{f}^{2}+\left( \gamma_{f}+\delta\right)^{2}}\#\left( S4 \right) \end{aligned}$$

where $\Delta\omega_{i}=\omega-\omega_{f}$.

Figure S4(b) shows the phase change $\Delta\varphi$ due to the redshift of the resonant frequency of a mirror-backed metasurface. To obtain larger phase change, the operating frequency will be in the range of the two resonant frequencies, i.e. $\omega_{f}<\omega_{a}<\omega_{i}$. Therefore, the reflection coefficient at $\omega_{a}$ before tuning will be in the top half plane, while the reflection coefficient after tuning will be in the bottom half plane. Further, based on our calculation of the radiative loss rate for various designs, we assume that the radiative loss rate decreases when then bias voltage increases, so that $\gamma_{i}>\gamma_{f}$, and the metasurface before tuning has to be in the overcoupled state, i.e. $\gamma_{i}>\delta$. Then, to achieve phase tuning larger than 270 degrees, the reflection before tuning should be in the 2nd quadrant, which means $0<\gamma_{i}^{2}-\delta^{2}<\Delta\omega_{i}^{2}$.

Figure S4(c) and (d) show the reflection trajectory and the phase change varying with the radiative loss rate. With decreasing the radiative loss and redshifting the resonant frequency, achieving a continuous large phase change necessitates that the metasurface remains overcoupled as the resonant frequency shifts to the operating frequency $\omega_{a}$, which is denoted as the intersection of the reflection trajectory (red curve in Figure S1c) with the positive real axis. Conversely, as the metasurface becomes undercoupled as the resonant frequency shifts to the operating frequency ($\omega_{a}=\omega_{0}$), the trajectory of the reflection vary with the radiative loss rate lies in the 2^nd^ and 3^rd^ quadrants, as shown as the blue curve in Figure S4(c). This results in a phase jump at the resonant frequency, as depicted by the blue curve in Figure S4(d), where the reflection trajectory crosses the real axis. Therefore, an overcoupling state for the metasurface, whose resonant frequency is at the operating frequency, is an indicator to achieve a continuous phase change over 180 degrees. Furthermore, the trajectory of the reflection has to cross the positive real axis. Then, the large phase change at $\omega_{a}$ can be described as

$$\begin{aligned} \Delta\varphi\left( \omega_{a} \right)=\left[ arctan\frac{2\gamma_{f}{\Delta\omega}_{f}}{\Delta\omega_{f}^{2}+\left( {\delta^{2}-\gamma}_{f}^{2} \right)}-\pi\right]-\left[ arctan\frac{2\gamma_{i}{\Delta\omega}_{i}}{\Delta\omega_{i}^{2}+\left( {\delta^{2}-\gamma}_{i}^{2} \right)}+\pi\right]\#\left( S5 \right) \end{aligned}$$

The maximum phase change can be obtained using the condition of $\frac{d\Delta\varphi\left( \omega_{a} \right)}{d\omega_{a}}=0$.

$$\begin{aligned} \frac{d\Delta\varphi\left( \omega_{a} \right)}{d\omega_{a}}=\frac{2\gamma_{f}\left[ \Delta\omega_{fa}^{2}+\left( \gamma_{f}^{2}-\delta^{2} \right) \right]}{\left[ \Delta\omega_{fa}^{2}+\left( \delta+\gamma_{f} \right)^{2} \right]\left[ \Delta\omega_{fa}^{2}+\left( \delta-\gamma_{f} \right)^{2} \right]} \\ -\frac{2\gamma_{i}\left[ \Delta\omega_{ia}^{2}+\left( \gamma_{i}^{2}-\delta^{2} \right) \right]}{\left[ \Delta\omega_{ia}^{2}+\left( \delta+\gamma_{i} \right)^{2} \right]\left[ \Delta\omega_{ia}^{2}+\left( \delta-\gamma_{i} \right)^{2} \right]}=0\#\left( S6 \right) \end{aligned}$$

where $\Delta\omega_{ia}=\omega_{a}-\omega_{i}$ and $\Delta\omega_{fa}=\omega_{a}-\omega_{f}$ If we apply another constraint that requires the reflection amplitude does not change significantly before and after tuning, then we have

$$\begin{aligned} \frac{\gamma_{f}}{\Delta\omega_{fa}^{2}+\left( \delta+\gamma_{f} \right)^{2}}\approx\frac{\gamma_{i}}{\Delta\omega_{ia}^{2}+\left( \delta+\gamma_{i} \right)^{2}}\# \left( S7 \right) \end{aligned}$$

And Equation S6 can be reduced as

$$\begin{aligned} \frac{\gamma_{f}-\delta}{\Delta\omega_{fa}^{2}+\left( \delta-\gamma_{f} \right)^{2}}\approx\frac{\gamma_{i}-\delta}{\Delta\omega_{ia}^{2}+\left( \delta-\gamma_{i} \right)^{2}}\#\left( S8 \right) \end{aligned}$$

From Equations S7 and S8, we can obtain

$$\begin{aligned} \Delta\omega_{ia}^{2}-\Delta\omega_{fa}^{2}\approx\left( \gamma_{f}-\delta\right)^{2}-\left( \gamma_{i}-\delta\right)^{2}\#\left( S9 \right) \end{aligned}$$

Generally, the frequency shift is much larger than the change of the radiative loss, that is $\left| \omega_{i}-\omega_{f} \right|\gg\left| \gamma_{i}-\gamma_{f} \right|$, then $|2\omega_{a}-\omega_{i}-\omega_{f}|\ll|\gamma_{i}+\gamma_{f}-2\delta|$, which indicates that the maximal phase change will be close to the arithmetic average of the two resonant frequencies, i.e. $\omega_{a}\approx(\omega_{i}+\omega_{f})/2$. Therefore, larger frequency shift could push the operating frequency $\omega_{a}$ closer to the negative real axis, leading to large phase change and large reflection amplitude as shown in Figure S4(b).

For metasurfaces operating in the microwave range, the material loss is generally negligible ($\delta\approx0$), allowing the metasurface to keep overcoupled during modulation. This results in easily achievable large phase tuning and large reflection amplitude. However, in the terahertz range, material loss from metals, substrate and liquid crystals becomes significant. Achieving large phase tuning and high reflection amplitude in this range requires meticulous design of the metasurfaces, which not only exhibit large radiative loss rates, but also can achieve significant frequency shift. As the operating frequency gets higher in the terahertz range, the loss tangents of most materials get higher, necessitating much larger radiative loss rates for designing metasurfaces. Therefore, a simple adaptation of metasurfaces designed for the microwave range is inadequate. Additionally, achieving large radiative loss rates with significant frequency shifts may require complex geometries beyond conventional metasurfaces. Such a design process for metasurfaces with metal-insulator-metal configurations using conventional parameter sweeping becomes highly involved. When additional structures, such as thick substrate/superstrate or multilayer metasurfaces, are included, the design space expands significantly, leading to more complex scattering spectra. Consequently, optimization design cannot be handled using traditional forward design methods. Novel design strategies have to be adopted.

1. Modified coupled mode theory

For a cascade system with two homogeneous layers stacked together in the air as shown in Figure 2, the total scattering can be described using the transfer matrix method:

$$\begin{aligned} M_{w}=M_{outr}M_{r}M_{rs}M_{sup}M_{outs}\#\left( S10 \right) \end{aligned}$$

where *M_r_* and *M_sup_* describe the scattering from bottom resonator and the superstrate, respectively; *M_outr_*, *M_rs_* and *M_out_* denotes the scattering through the interface between the resonator and the bottom air, the interface between the resonator and the superstrate, and the interface between the superstrate and the air.

In our model, we only focus on the reflection *S_t11_* from the top surface of the superstrate, which can be derived as:

$$\begin{aligned} r_{t,s}\left( \omega\right)= \\ \frac{\frac{j({n_{1}}^{2}-{n_{2}}^{2})sin(n_{2}k_{0}d)}{4n_{1}n_{2}t_{m}}+r_{m}\frac{2n_{1}n_{2}\cos\left( n_{2}k_{0}d \right)-j\left( {n_{1}}^{2}+{n_{2}}^{2} \right)\sin\left( n_{2}k_{0}d \right)}{4n_{1}n_{2}t_{m}}}{\frac{{j(n_{1}}^{2}+{n_{2}}^{2})sin\left( n_{2}k_{0}d \right)+2n_{1}n_{2}\cos\left( n_{2}k_{0}d \right)}{4n_{1}n_{2}t_{m}}-r_{m}\frac{j\left( {n_{1}}^{2}-{n_{2}}^{2} \right)\sin\left( n_{2}k_{0}d \right)}{4n_{1}n_{2}t_{m}}}\#\left( S11 \right) \end{aligned}$$

where the subscript ‘*t*’ denotes the total reflection from the top surface and ‘s’ indicates the reflection at ‘On/Off’ states of the dynamic metasurface; *t_m_* represents the transmission from the bottom of the metasurface to the top, which is close to zero due to the metal-backed metasurface in our design; *r_m_* describes the reflection from the top surface of the metasurface; *n_1_* is the refractive index of air; *n_2_* is the refractive index of the superstrate and *d* is the thickness of the superstrate; *k_0_* is the wavenumber in the freespace. Through further simplification, Equation S11 can be further simplified as:

$$\begin{aligned} r_{t,s}(\omega)=\frac{-j+r_{m}(\eta-j\xi)}{(\eta+j\xi)+jr_{m}}\#\left( S12 \right) \end{aligned}$$

where

$$\eta=\frac{2n_{1}n_{2}}{n_{2}^{2}-n_{1}^{2}}\cot\left( n_{2}k_{0}d \right)$$

$$\xi=\frac{n_{2}^{2}+n_{1}^{2}}{n_{2}^{2}-n_{1}^{2}}$$

Next, we describe the verification process of Equation S12. During the process, the thickness of the superstrate is set as a constant such that the factors $\eta$ and $\xi$ in Equation S12 do not change. Given the symmetry of the metasurface, the resonant responses of the metasurface layer can be considered as Lorentzian responses

$$\begin{aligned} r_{m}\left( \omega\right)=-1+\frac{2\gamma_{1}}{j\left( \omega-\omega_{1} \right)+\left( \delta_{1}+\gamma_{1} \right)}+\frac{2\gamma_{2}}{j\left( \omega-\omega_{2} \right)+\left( \delta_{2}+\gamma_{2} \right)} \#\left( S13 \right) \end{aligned}$$

In Equation S13, we assume that there will be two resonances affecting the reflection in the monitored spectrum range. $\omega_{i}$, $\gamma_{i}$ and $\delta_{i}$ ($i=1,2$) are the mode frequency, radiative loss rate, and the intrinsic material loss rate for the first and second modes, respectively. Then these mode parameters can be easily fitted using Equation S12 using numerically simulated reflection spectrum $r_{t,s}(\omega)$. We have to mention that, only the reflection amplitude is used for parameter retrieval. Figure S5 display two comparison of the fitting and the simulation results for two different structures.

Figure S5. The accuracy verification of MCMT applying to two different structures. The solid line represents the electromagnetic simulation results and the dashed line represents the fitting results of MCMT formula. (a) MCMT formula fitting the deformed resonance curve of sample 1 which is strongly influenced by the substrate. (b) MCMT formula fitting the resonance curve of sample 2 with correspondingly less influenced by the substrate. Comparison of the unwrapped phase and phase difference between simulation and MCMT calculated are shown in (c) and (d), corresponding to sample 1 and sample 2 respectively. (e) The table of actual fitted physical parameters according to MCMT formula. The insets in the figure are the structures of the metasurface corresponding to the spectrum.


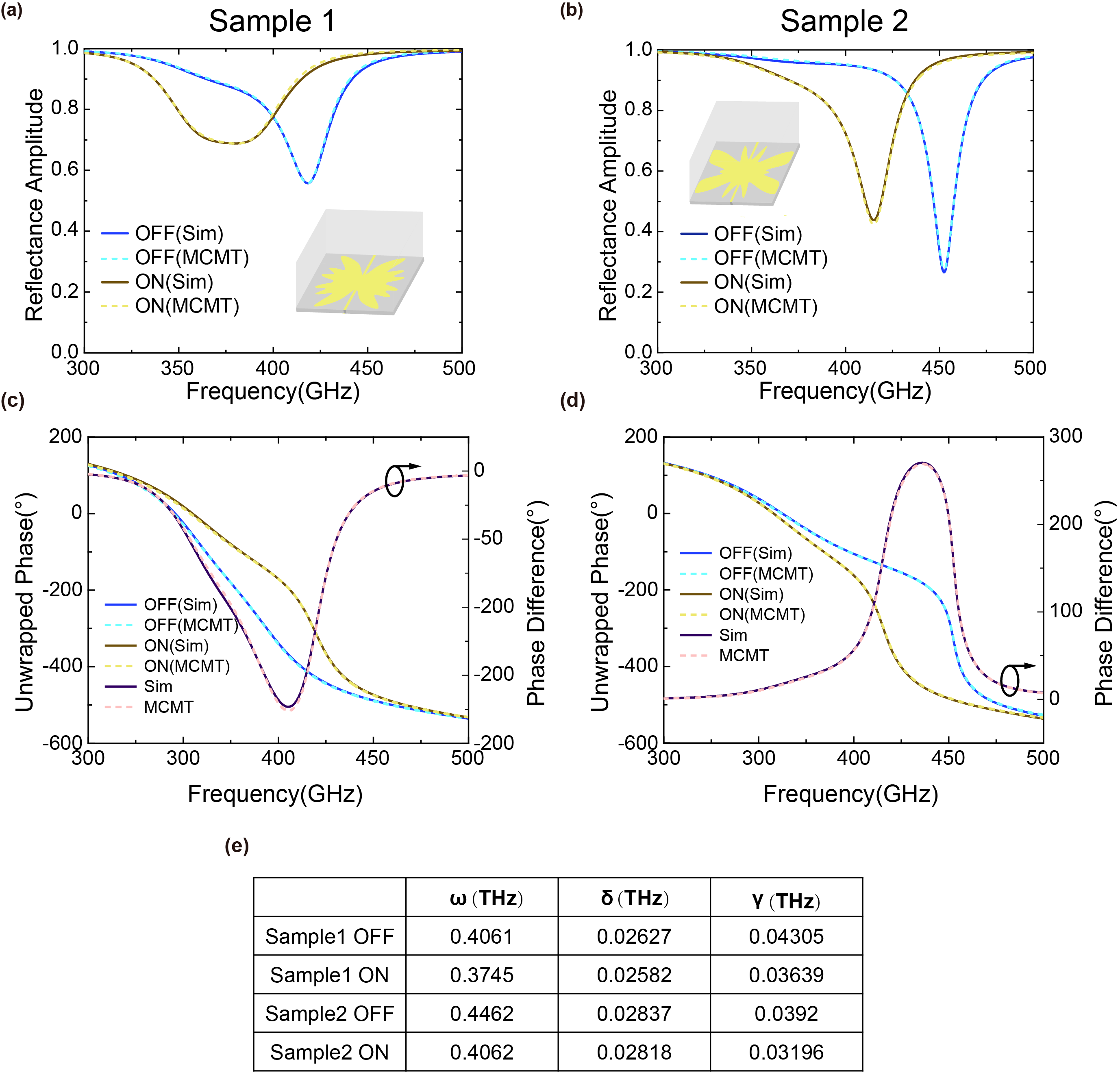


We use the MCMT formula to fit the reflection amplitude of two different structures, as shown in Figure S5(a) and (b). By comparing the formula fitted results with the reflectance amplitude spectrum obtained by electromagnetic simulation, it can be found that the proposed MCMT formula can achieve a high accuracy with the MSE all on the order of 10^-5^ (MSE of Sample 1 in ‘OFF’ and ‘ON’ state are 1.89*10^-5^ and 3.46*10^-5^ and that of Sample 2 are 1.55*10^-5^ and 3.46*10^-5^, respectively). We have to mention that, the fitting was only performed based on the amplitude spectra, the phase spectra can be predicted using the fitted MCMT parameters. To validate the model, we also compare the differences between the simulations and the predicted phase through using MCMT for two structures, as shown in Figure S5(c) and (d), respectively. The very good agreement of the curves indicates that our proposed MCMT formula is capable of effectively describing the physical properties of resonant structures affected by the superstrate.

1. Evaluation and discussion of intersection points with maximum phase difference

In this section, we will show that the intersection point of the On/Off reflection spectra is close to the midpoint of the two resonant frequencies for the Lorentzian resonators. To simplify the calculation, only the fundamental mode is considered. The intersection point of the reflection spectra of the Lorentzian resonator before and after the bias on can then be calculated as:

$$\begin{aligned} \left| r_{m}^{On}\left( \omega_{on} \right) \right|^{2}-\left| r_{m}^{Off}\left( \omega_{off} \right) \right|^{2}=\frac{4\delta_{on}\gamma_{on}}{\left( \delta_{on}+\gamma_{on} \right)^{2}+\left( \omega_{cp}-\omega_{on} \right)^{2}} \\ -\frac{4\delta_{off}\gamma_{off}}{\left( \delta_{off}+\gamma_{off} \right)^{2}+\left( \omega_{cp}-\omega_{off} \right)^{2}}=0\#\left( S14 \right) \end{aligned}$$

where $\omega_{cp}$ is the frequency of the intersection point, and $\delta_{on/off},\gamma_{on/off},\omega_{on/off}$ represents material loss, radiative loss and resonance frequency with the bias on and off, respectively. In our model, we assume the resonance is strong such that $\omega_{on/off}>>\delta_{on/off}$ and $\omega_{on/off}>>\gamma_{on/off}$. Then, the solution of Equation S14:

$$\begin{aligned} \omega_{cp}\approx\frac{\delta_{on}\gamma_{on}\left( \delta_{off}^{2}+\gamma_{off}^{2}+\omega_{off}^{2} \right)-\delta_{off}\gamma_{off}\left( \delta_{on}^{2}+\gamma_{on}^{2}+\omega_{on}^{2} \right)}{2\left( \delta_{off}\gamma_{off}\omega_{on}-\delta_{on}\gamma_{on}\omega_{off} \right)}\#\left( S15 \right) \end{aligned}$$

Due to limited change of material loss and radiative loss, if the condition $\gamma_{on} \approx\gamma_{off}$ and $\delta_{off}\approx\delta_{on}$ is considered, here we could get an easy approach to achieve $\omega_{cp}$, the form of which is as follows:

$$\begin{aligned} \omega_{cp}\approx\frac{\omega_{on}+\omega_{off}}{2}\#\left( S16 \right) \end{aligned}$$

Although this intersection point is not the same as the intersection point of the total reflection spectra with the superstrate included (when $\left| r_{t,s}^{On}\left( \omega_{on} \right) \right|^{2}=\left| r_{t,s}^{Off}\left( \omega_{off} \right) \right|^{2}$), our statistical analysis indicates that the intersection point of the resonator does not deviate from that of the combined structure. Therefore, we will use the midpoint of the two Lorentzian modes as the initial value for searching the mode parameters. Such a setting allows a fast gradient decent searching for the target operating frequency. Here we set $\omega_{on}= \omega_{t}-\Delta\omega/2$ and $\omega_{off}= \omega_{t}+\Delta\omega/2$ as the initial value of iterative optimization, which is helpful to quickly update and obtain required $\omega_{on}$ and $\omega_{off}$ during the process of iterative optimization from the targets.

Next, we show that the frequency where the maximum phase shift is achieved is also close to the intersection point the two reflection spectra of the combined structure. Instead of deriving such a relation from the Equation 5, Figure S6 shows the deviation of the two frequencies based on randomly generated 467 samples with maximum phase change over 220° in the frequency range of 0.3-0.5 THz. It is obvious that the frequency difference is within a few gigahertz.


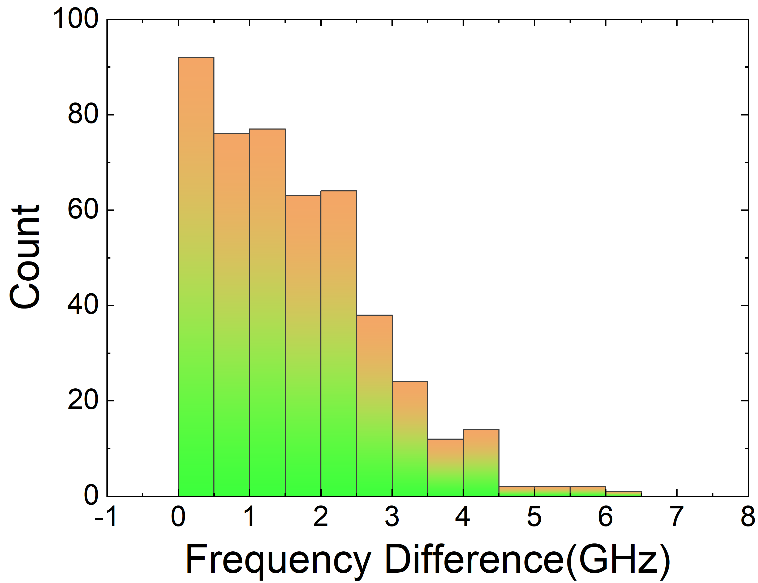


Figure S6. Statistical histogram of the difference between the frequency with the maximum phase difference and the intersection of the reflection spectra

1. Inverse design procedure

As depicted in Figure S7, the entire process can be delineated into two distinct parts: iterative optimization for determining the optimal physical parameters and inverse design for attaining the desired metasurface structure. In the initial step, physical parameters are randomly initialized within the range of 0 to 1. Leveraging the MCMT formula with continuously differentiable characteristics, the physical parameters conducive to achieving the design objectives are swiftly acquired through iterative optimization employing the gradient descent method. The loss function employed utilizes the squared deviation between the actual and target phase differences. Following the completion of iterative optimization via gradient descent, the second target reflectance amplitude at the operating frequency is evaluated. Typically, the primary objective of the design process is to maximize the reflection amplitude. However, the ability to achieve a large phase difference while simultaneously enhancing reflection amplitude is constrained by the limited range of resonant frequency shift. Therefore, establishing reasonable and appropriate amplitude evaluation criteria is instrumental in realizing the anticipated device performance.


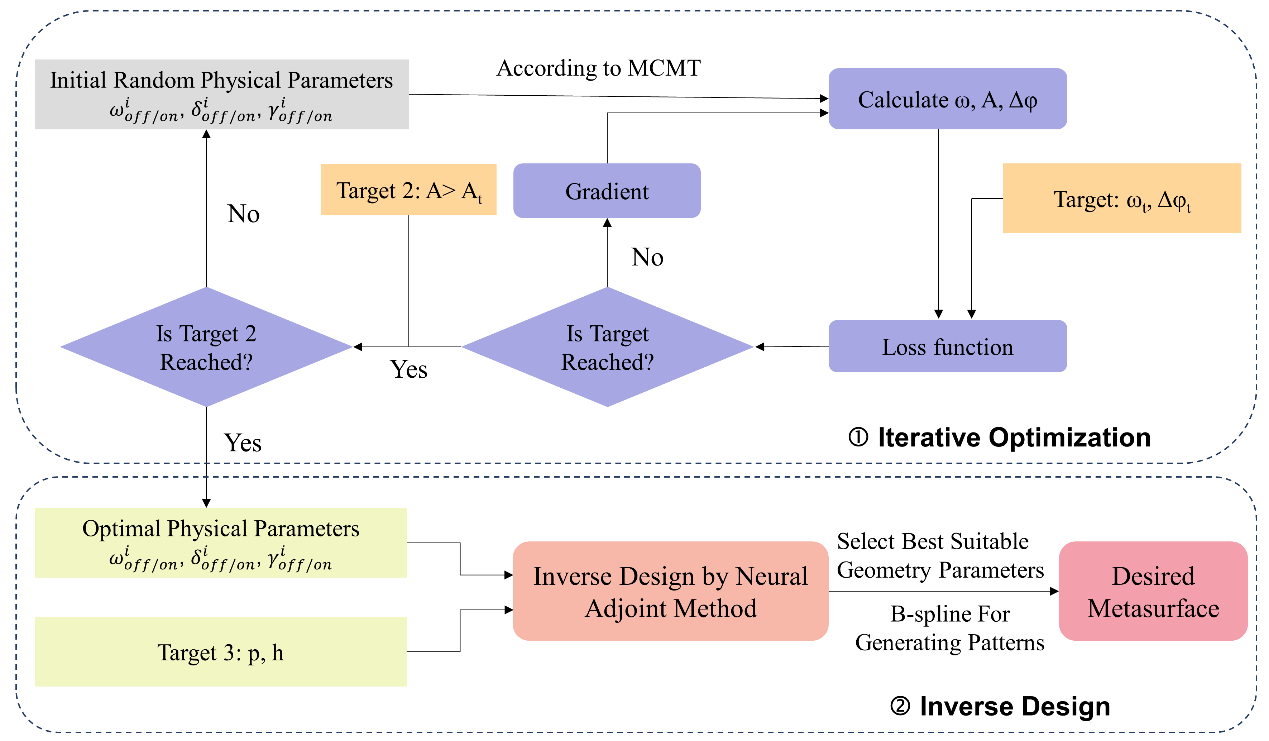


Figure S7. Design procedure of metasurface using physical-formula-based iterative optimization and NA-based inverse design

Upon obtaining the target physical parameters of the device through iterative optimization, measures are taken to ensure strong alignment with the practical manufacturing process. Specifically, the crystal cell period and the thickness of the liquid crystal layer were chosen as additional objectives, further constraining the number of viable solutions. Ultimately, the desired metasurface configuration is achieved through inverse design and the B-spline algorithm. The considerable design flexibility afforded by the resonant metal layer ensures a vast design space for the device, facilitating the computation and exploration of multiple geometric parameter solutions that fulfill the design objectives.

1. Detailed explanation of forward and backward loss functions in ResMLP network

The loss function of the forward training procedure is shown as:

$\begin{aligned} L=\left( s-\hat{f}\left( \hat{g} \right) \right)^{2}+\alpha*\sum_{i=1}^{2} ReLU\left( \omega_{max}-\omega_{0i,s} \right)\#\left( S17 \right) \end{aligned}$

Where $s$ and $\hat{f}(\hat{g})$ are the target spectrum and predicted spectrum respectively. $\omega_{max}$ is the critical resonant frequency and $\omega_{01,l}, \omega_{02,l}$(corresponding to ‘OFF’ and ‘ON’ state respectively) represent the resonant frequency of higher-order modes. $\alpha$ is the hyperparameter. The first term on the right is the Mean Squared Error (MSE) between the spectra based on MCMT using the target and the predicted mode parameters. The second term is defined to establish a specific order during training for the basic mode and higher-order mode during output. $\alpha$ is found to be 20 for better discrimination. This loss term guarantees the order of the predicted mode parameters.

Figure S8. (a) Detailed whole structure of Traditional MLP network and Residual MLP network. Both training loss (b) and validation loss (c) curves exhibit the faster convergence of Residual MLP (green line) than traditional MLP (pink line).


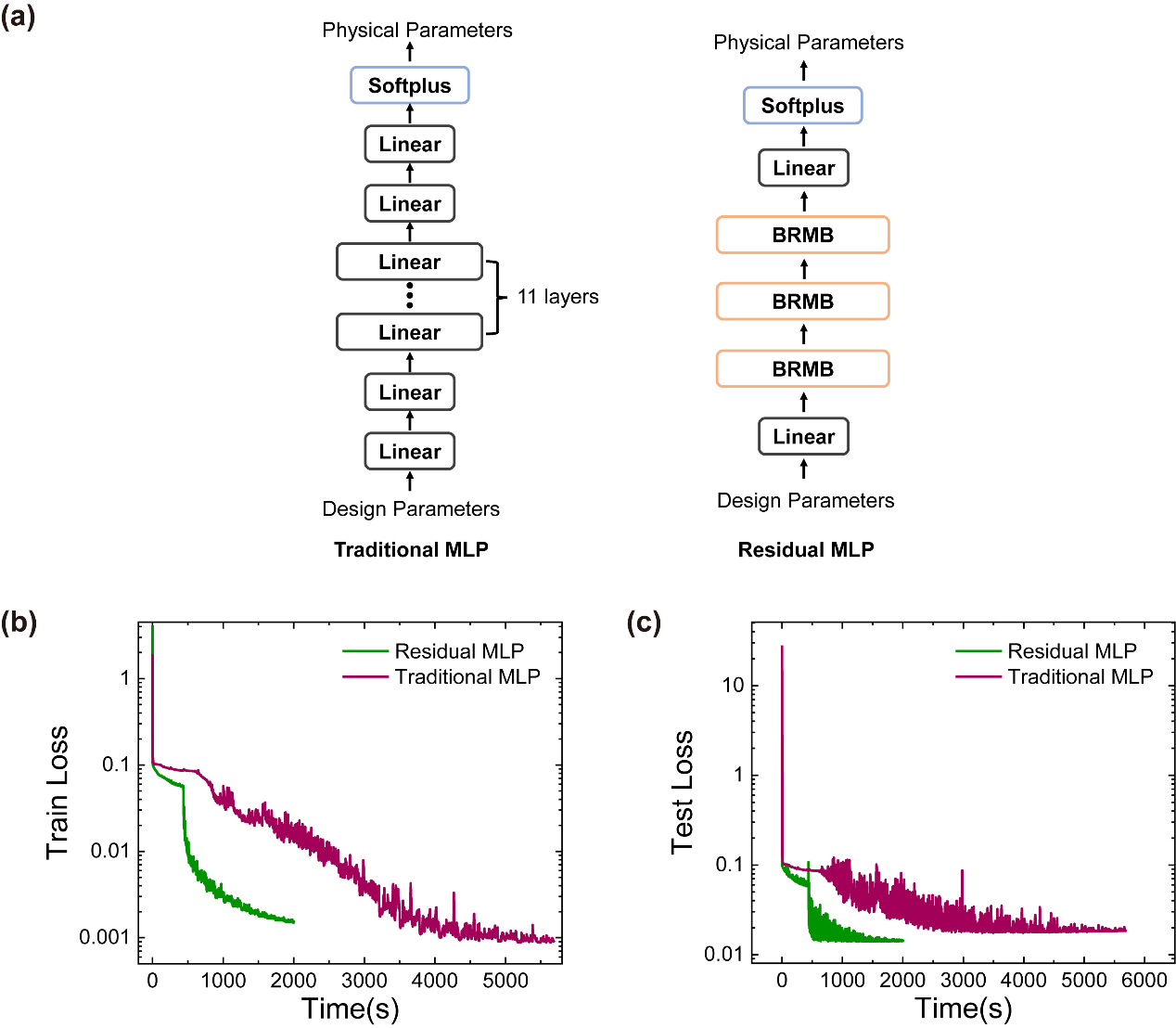


The neural adjoint method emerges as a powerful tool for inverse design. Specifically, the initial step involves the fixation of all weights and biases associated with trained Residual MLP network. Subsequently, only the gradient of the forward model with respect to the network (i.e., geometry) input is exclusively calculated, and the optimal inverse solution is found through iterative optimization of randomly initialized geometric values [2]. Notably, our focus extends to the refinement and specificity of the loss function in the design process:

$L=\sum_{m=1}^{2} \sum_{i=1}^{2} \alpha_{m}\left[ \left( f_{ti}^{m}-f_{i}^{m} \right)^{2}+\left( \delta_{ti}^{m}-\delta_{i}^{m} \right)^{2}+\left( \gamma_{ti}^{m}-\gamma_{i}^{m} \right)^{2} \right]+10* ReLU\left( \left| \hat{g}-\mu_{g} \right|-\frac{1}{2}R_{g} \right)+\beta*\left( \left| p-p_{t} \right|+\left| h-h_{t} \right| \right)$ $\left( S18 \right)$

where $m$ equals to '1' and '2' representing the basic mode and higher-order mode, meanwhile $i$ takes on the values '1' and '2,' signifying the 'OFF' and 'ON' states, respectively. ‘t’ denotes the target. In the first term, hyperparameters $\alpha_{1}$ and $\alpha_{2}$ are set to be 10 and 0.1, respectively. Such a setting ensures a significant emphasis on the basic mode during inverse design procedure. The second term represents the boundary loss function, encapsulating the requirement that the solution does not deviate beyond the specified feasible domain. Finally, the third term introduces constraints for the inverse design of devices concerning their period $p$ and liquid crystal layer thickness $t_{lc}$. The hyperparameter $\beta$ is empirically set to 0.01.

We conducted experimental comparisons on the performance of traditional MLP and Residual MLP. The traditional MLP network adopts a fully connected approach connecting 15 hidden layers. The number of neurons in the first two hidden layers and the last two hidden layers is 2000, while the number of neurons in the remaining 11 middle layers is 4000. Each hidden layer sequentially contains linear layers, batchnorm layers, and the activation function is Linear rectification function (Relu). On the other hand, The Residual MLP network consists of two linear layers in the front and back, along with three BRMB modules in between. For each BRMB module, a bottleneck design is employed in its two internal linear layers—The input size for the first layer is 4096, with an output size of 512, whereas the input size for the second layer is 512, with an output size of 4096. Softplus activation function is applied to the final output in both two networks, ensuring non-negative network outputs. Detailed information for both two networks is shown in Table S1, and whole structures of the two networks are illustrated in Figure S8(a).


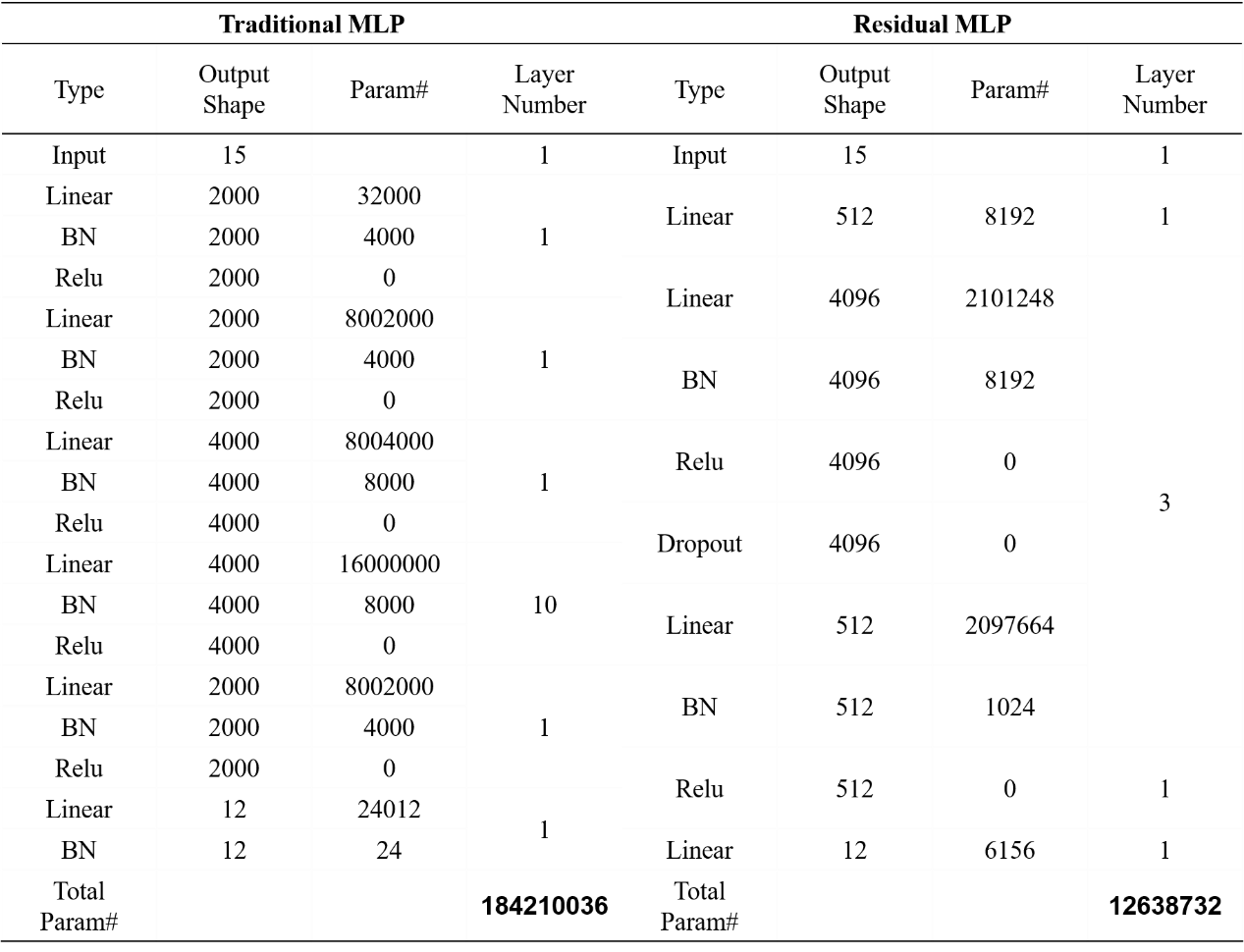


Table S1: Detailed information of every single layer in both two networks

As shown in Figure S8(b-c), it is evident from the training and validation errors that Residual MLP exhibits significantly faster convergence than its traditional counterpart. Moreover, it attains a smaller optimal validation set error upon convergence. Due to the optimization of the network architecture, similar acceleration effects are observed in inverse design as well.

1. Inversely designed reconfigurable metasurface by PIID

To obtain a dynamic metasurface with maximum phase change as large as 300^o^, several demands for the inverse design will be given: phase difference $\Delta\varphi_{t}=$ 300° and $A_{t}\geq$ 0.8 at a working frequency $\omega_{t}=$0.435THz. Besides, the target period and LC layer thickness are set to 240μm and 10μm, respectively. The target mode parameters are first obtained through the adaptive gradient descent method. As is shown in Figure S9, the phase difference spectrum and two reflectance spectra calculated by MCMT:


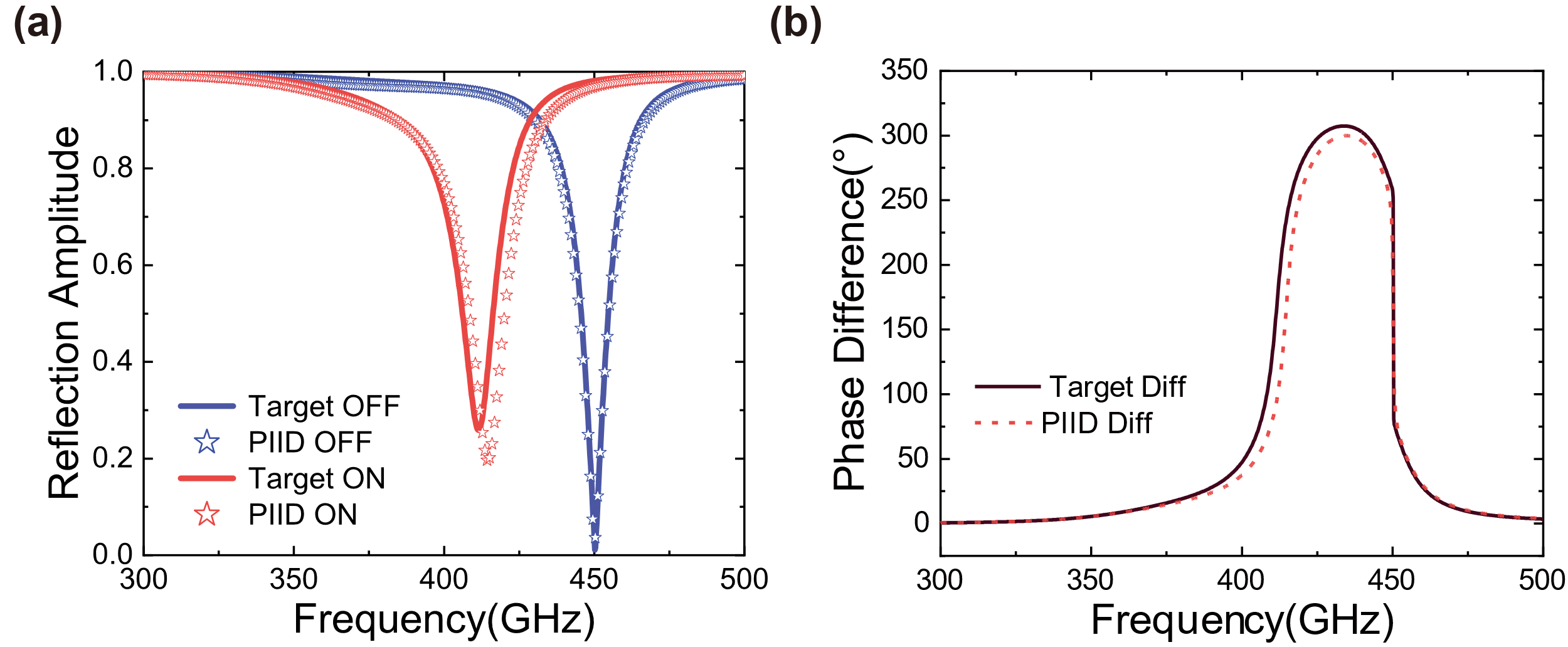


Figure S9. (a) Comparison of target reflectance spectra and PIID generated reflectance spectra (b) Comparison of phase difference spectra generated by target and PIID

using target mode parameters are all match with the spectra through the inversely designed structure by PIID. The generated geometry is also shown in Figure 2c. Finally, a phase difference of 300 ° has been generated at the target frequency point 435GHz. The period and LC layer thickness of the designed structure are completely consistent with demands. The generated metasurface geometries illustrated in the main text are shown in Table S2.


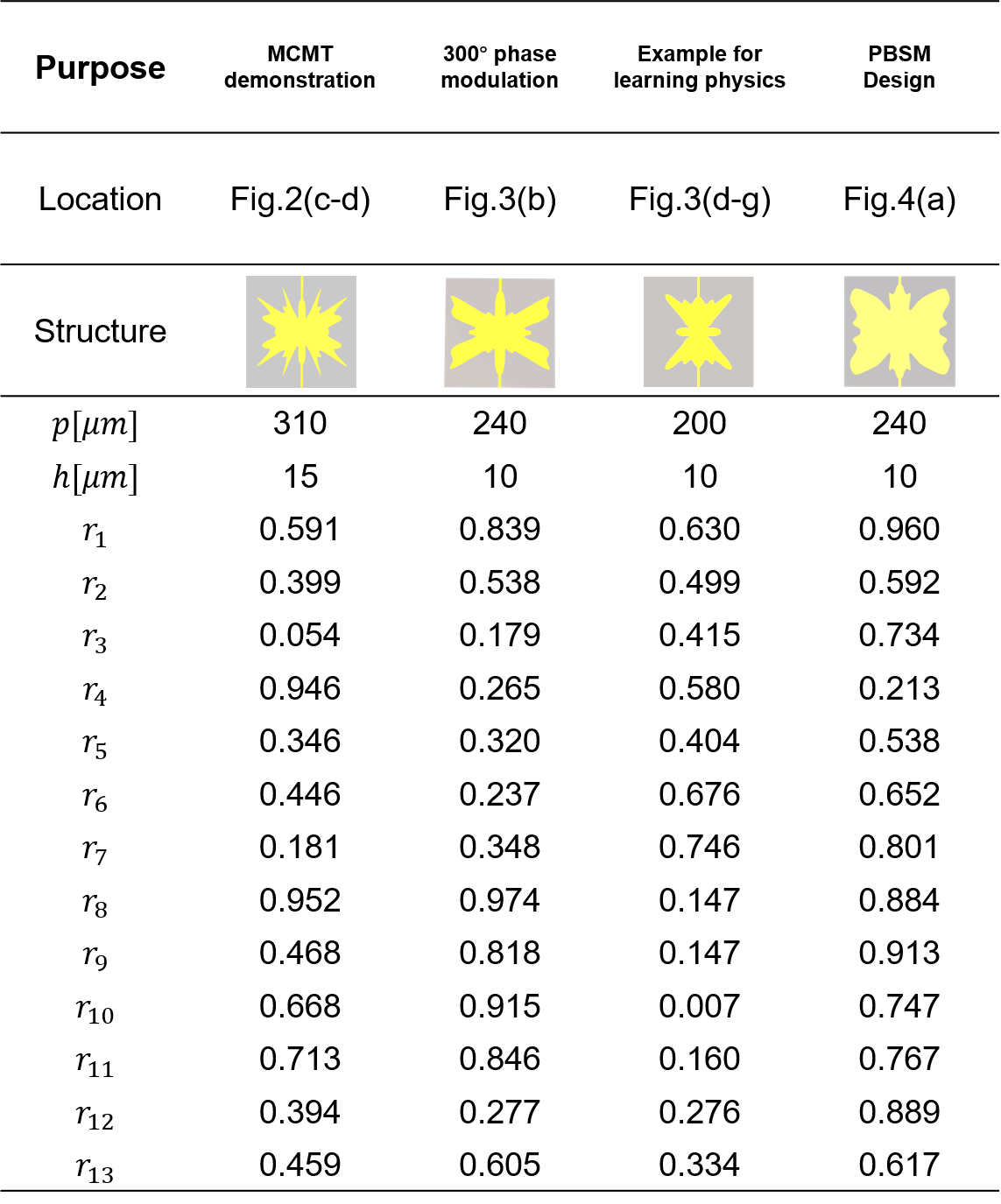


Table S2. Geometric parameters of all structures mentioned in the main text

1. Physics learned from the ResMLP network

Here we utilized the trained Residual MLP neural networks to predict the relationship between the mode loss rate and the geometry parameters, as shown in Figure S10. With the increasing of the LC height, the material loss rate $\delta$ decreases while the radiative loss rate $\gamma$ increases, therefore this is an effective method to alter the device to operate at under coupled condition. Besides, the material loss rate is not sensitive to the period according to the prediction. Through comparing the NN prediction and simulation results, we could further validate the learned physical.


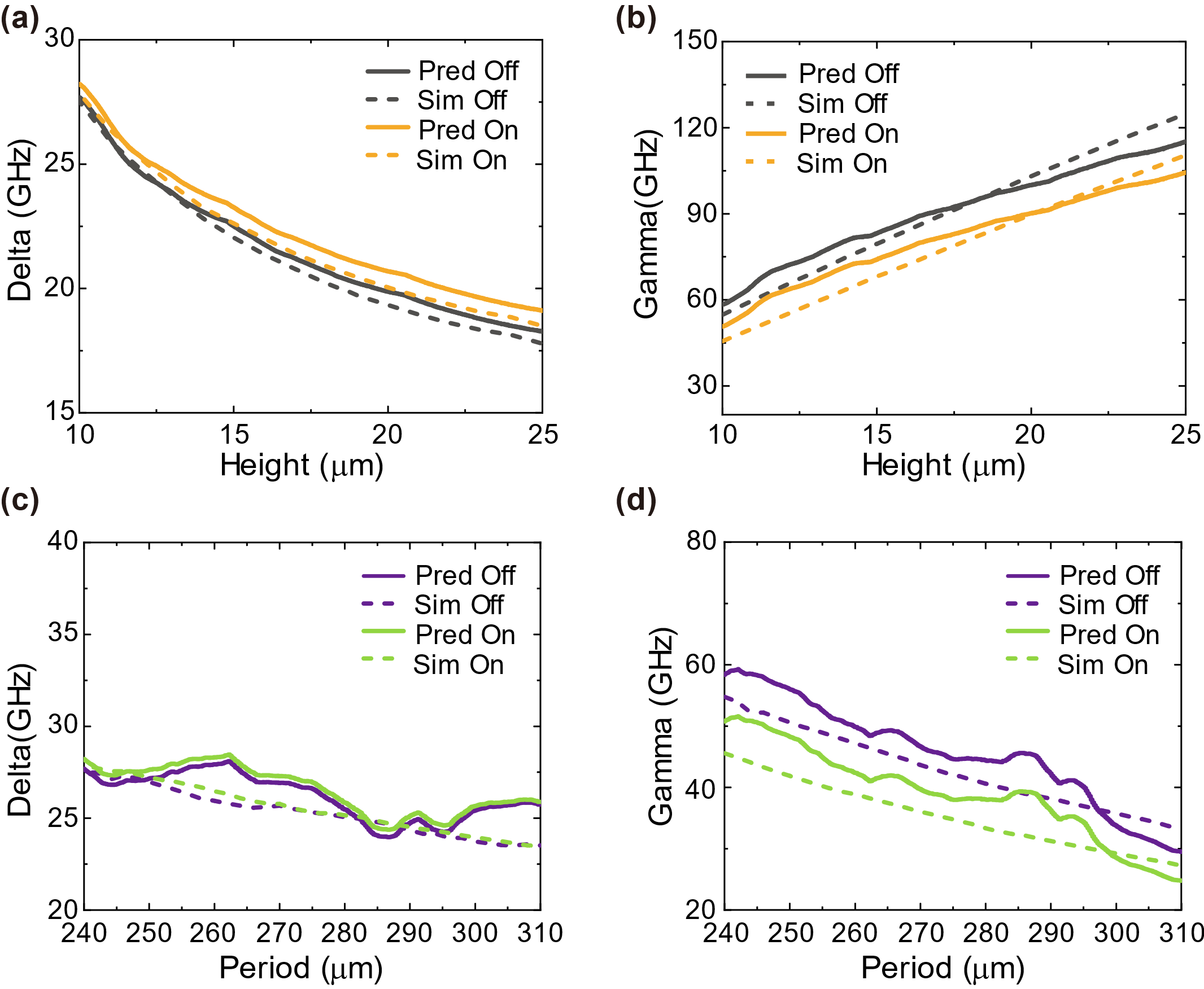


Figure S10. Comparison of the NN-predicted and simulated relationship between (a) material loss and the LC height (b) radiative loss and the LC height (c) material loss and the period (d) radiative loss and the period

1. The relationship of geometry and mode parameters

For the portion involving network predictions, we kept all other structural parameters fixed and only varied the value of a single parameter. The altered geometric parameters were then input into the trained neural network to predict the corresponding mode parameters.

To determine whether the network's predictions could serve as a surrogate model for electromagnetic simulations, we conducted electromagnetic simulations on structures with the same geometric parameters. After obtaining the reflection spectrum ($r_{i}\left( \omega\right)$ in Equation 2) from the simulations, we used the MCMT formula to fit this reflection spectrum. Specifically, to simplify the fitting process, we first removed the influence of the substrate by calculating the fixed constants $\eta$ and $\xi$ as described in Equation 2. This enabled us to compute $r_{i}^{m}\left( \omega\right)$, a reflection spectrum that exhibits a nearly pure Lorentzian resonance. By applying the least squares method to fit $r_{i}^{m}\left( \omega\right)$ with the Lorentzian formula, we obtained the desired modal parameters $\{\omega_{0}, \gamma, \delta\}$.

Our network can predict the changes in modal parameters for both the OFF and ON states of a single structure. Therefore, we performed electromagnetic simulations and comparisons for devices in both states to verify that the dynamic metasurface properties could be accurately predicted by the trained neural network. This not only demonstrates that the network can serve as a surrogate model for electromagnetic simulations but also indicates that the parameters learned by the network have significant physical meaning and interpretability.

1. Multiple encoding schemes (2-bit, 1-bit and ‘tri-states’) for THz beam steering

In addition to the 2-bit coding sequencies discussed in the main text, the THz beam deflection performances with other 2-bit coding sequencies applied to the metasurface are shown in Figure S11.


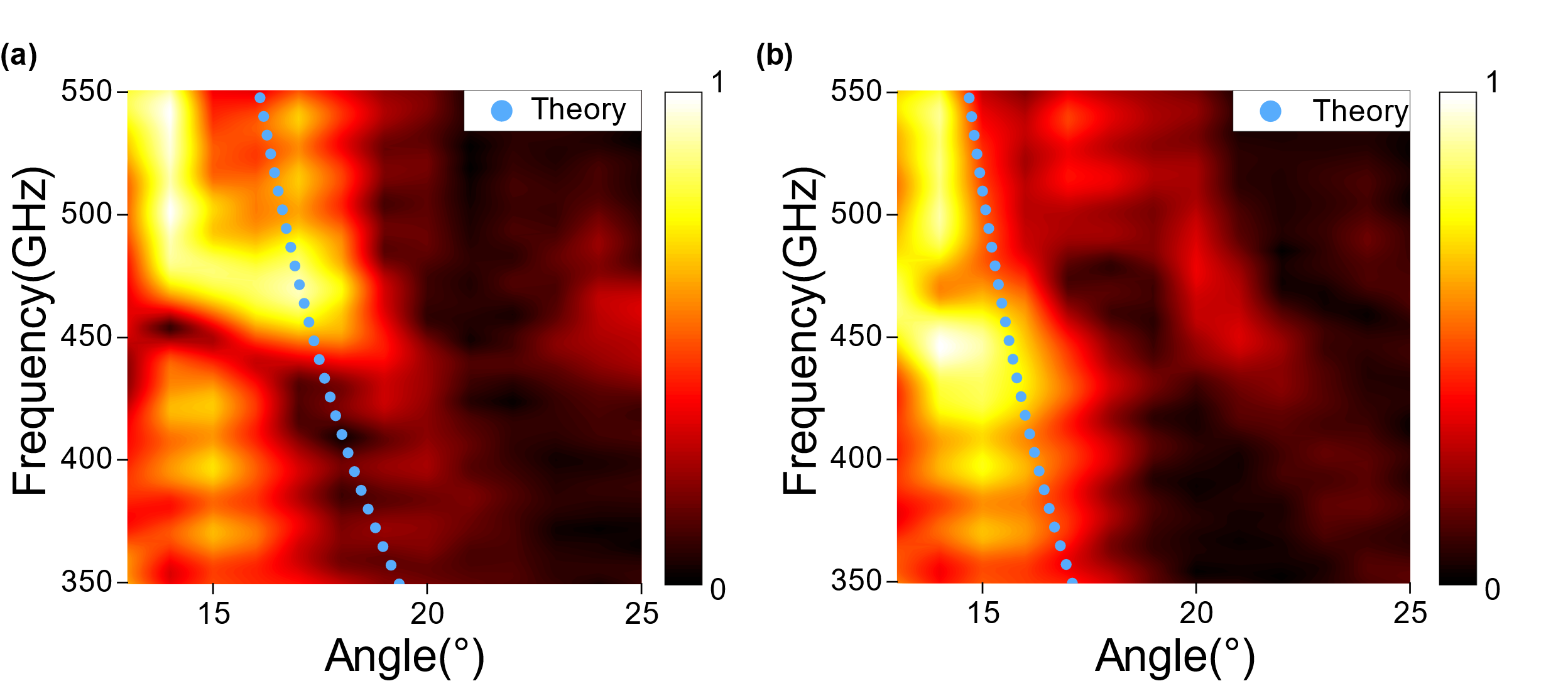


Figure S11. Measurements of the beam deflection with the metasurface encoded with different sequencies (a) /00 00 00 01 01 01 10 10 10 11 11 11/ (b) /00 00 00 00 01 01 01 01 10 10 10 10 11 11 11 11/ The hollow circle curve represents theoretical calculated deflection angles.


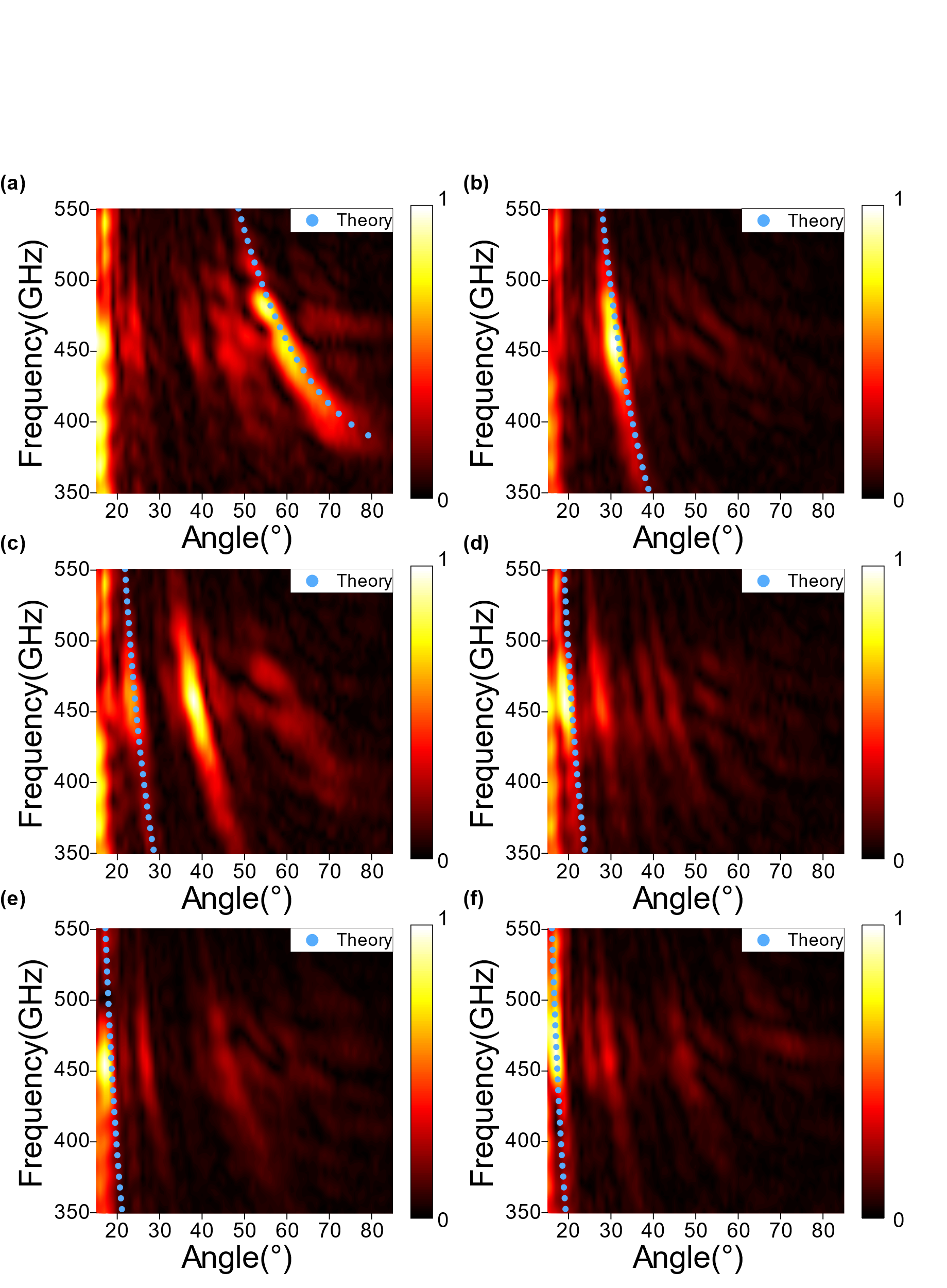


Figure S12. Measurements of the beam deflection with the metasurface encoded with the coding periods (a) /00 11/ (b) /00 00 11 11/ (c) /00 00 00 11 11 11/ (d) /00 00 00 00 11 11 11 11/ (e) /00 00 00 00 00 11 11 11 11 11/ (f) /00 00 00 00 00 00 11 11 11 11 11 11/. The dotted curves represent theoretically calculated deflection angles.

Here we attempt to explore a new coding sequence that can be loaded to the metasurface. Instead of applying 0 V and 8V to achieve phase modulation of 180^o^ at the frequency of 465.2 GHz, we encode the two different metasurface column with 0V and 15 V respectively. Then the 180^o^ phase change can be achieved at 451 GHz and 477 GHz simultaneously, thereby eliciting beam deflection effects at these two frequencies. As illustrated in Figure S12 (a), experimental evaluation reveals beam deflection effects centered around two distinct frequency points: 453 GHz and 483 GHz, respectively.


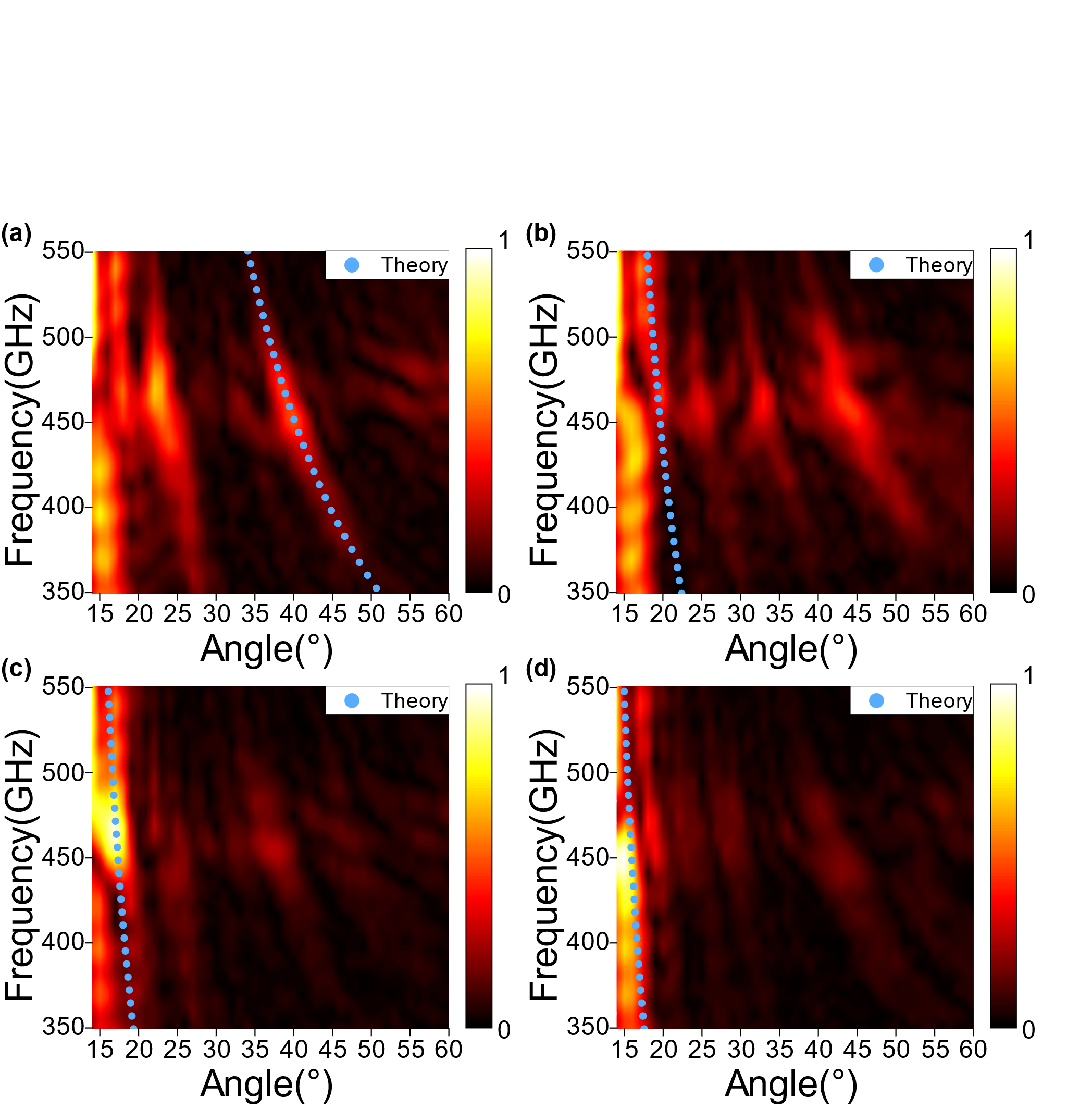


Figure S13. Measurements of the beam deflection with the metasurface encoded in ‘tri-state’ sequence with the coding periods (a) /0 1 2/ (b) /0 0 0 1 1 1 2 2 2/ (c) /0 0 0 0 1 1 1 1 2 2 2 2/ (d) /0 0 0 0 0 1 1 1 1 1 2 2 2 2 2/. The hollow circle curve represents theoretical calculated deflection angles.

As the encoding cycle continues to lengthen (Figure S12(b-f)), although the beam deflection effects of the two frequency points are gradually merged, the overall frequency range of effective beam deflection effect is expanded to a certain extent.

Further, we also tested the beam deflection performance of the metasurface operating under the 'tri-state', as shown in Figure S13. The implementation of this coding scheme can be entirely attributed to the realization of large phase differences during the transition from 'OFF' to 'ON' states. After achieving phase differences of 0°, 120°, and 240° respectively (corresponding to the '0', '1', and '2' states), the beam deflection effect was experimentally tested under four kinds of 'tri states' encoding periods (except the coding period /0 0 1 1 2 2/ mentioned in the main text), and it showed good consistency with the theoretical curves. It is noteworthy that discrepancies between the observed deflection phenomenon and the theoretical model primarily stem from the dispersion of deflection energy induced by higher-order diffraction effects.

1. Asymmetric beam deflection energy distribution of the device with 2-bit programmable capability

According to the discussion in the main text, m=-1 represents the beam generated by specular reflection, and m=0 and m=-2 represents the split deflection beam along the x-axis forward and backward, respectively. Both of the angles between their deflection direction and the normal direction are exactly the same, while the reflectance amplitude is not consistent, which is the main difference between 2-bit encoding and 1-bit encoding. As depicted in Figure S14, it is observed that the positive gradient direction (m=0, also the pre-designed angle) exhibits superior deflection efficiency compared to the anti-gradient direction (m=-2). This suggests that the metasurface endowed with 2-bit programmable capability allocates a greater proportion of energy to the beam in the intended deflection direction, surpassing the performance of 1-bit programmable device.


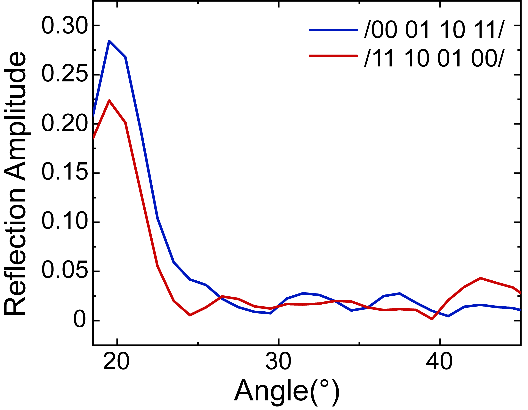


Figure S14. Experiment results of the reflectance amplitude when the device is encoded with the coding period /00 01 10 11/ and /11 10 01 00/. The deflection beam representing the diffraction order m=0 has higher reflectance amplitude 0.284 than the deflection beam of diffraction order m = -2 with a reflection amplitude of 0.224 in theoretical angle 21° at 462GHz.

1. Measurements of high-order diffractions of the programmable metasurfaces

As described in Equation 4, different $m$ values represent different diffraction orders M. The case of $m=0$ is discussed in the main text, while the phenomenon of high-order diffraction can also be measured in practice, as shown in Figure S15. For the observed frequency of 462 GHz, the theoretical calculated diffraction angle is close to the actual test deflection angle, and the diffraction phenomenon is more obvious when M is 1,2,4 than that when M takes the value of 3. In addition, by the comparison between the diffraction phenomenon of the fabricated device encoded with the coding period /00 00 01 01 10 10 11 11/ and that of the device encoded with the anti-coding period /11 11 10 10 01 01 00 00/, it is clear that energy distribution of the split deflection beam is inconsistent under the positive coding sequency and the anti-coding sequency, which is also another difference comparing to 1-bit coding programmable device.


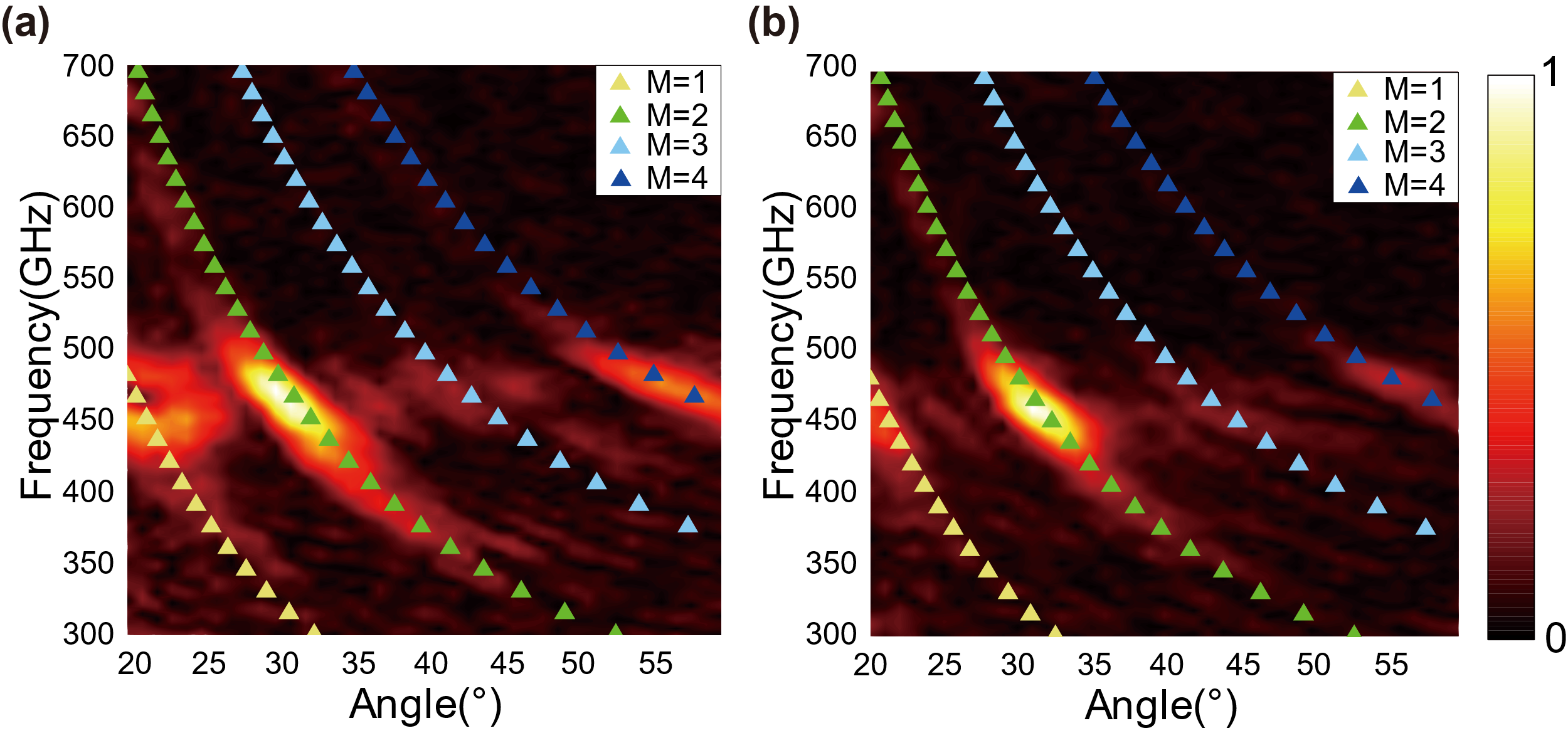


Figure S15. Measurements of higher orders M=1,2,3,4 of the device. (a) The device encoded with the coding period /00 00 01 01 10 10 11 11/. (b) The device encoded with the anti-coding period /11 11 10 10 01 01 00 00/. The theoretical calculation curves of different diffraction orders are shown in the form of discrete triangle points.

1. The simulation results of the beam steering performance

Numerical simulations have been conducted on the programmable metasurface employing various encoding schemes, as shown in Figure S16. Electromagnetic waves are incident at 0° on the device, and the spatial electric field distribution is exhibited at 460GHz (1bit), 455GHz (2bit), and 455GHz (tri-state), respectively. It is discernible that compared to beam splitting with nearly identical deflection efficiency achieved by a 1-bit encoder device, a 2-bit encoder device can concentrate the energy of beam deflection relatively more effectively in the desired direction while efficiently suppressing signals expected to undergo vertical reflection. Furthermore, in the case of tri-state encoding, spatial asymmetry in beam deflection persists. The theoretically calculated main lobe deflection angles of 19.86°, 20.08°, and 27.25° align closely with the observed deflection angles obtained through simulation.


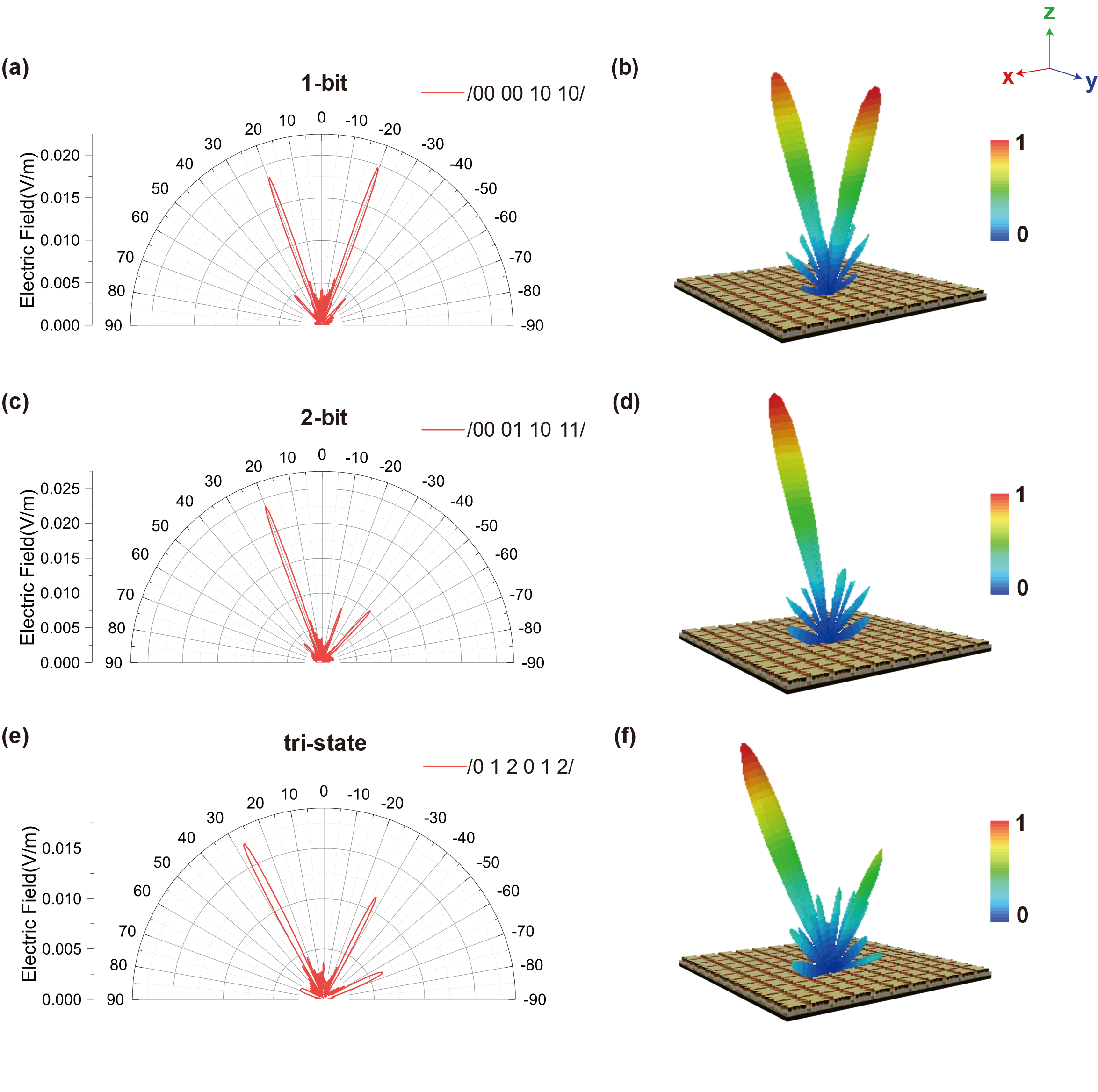


Figure S16. Simulated 2D and 3D spatial electric field pattern of the beam steering performance corresponding to the metasurface encoded with (a-b) \00 01 10 11\, (c-d) \00 00 10 10\, (e-f) \0 1 2 0 1 2 0 1 2\, meanwhile representing the different encoding capability of 2-bit, 1-bit and tri-state respectively.

1. Performance comparison of various devices in Terahertz beamsteering


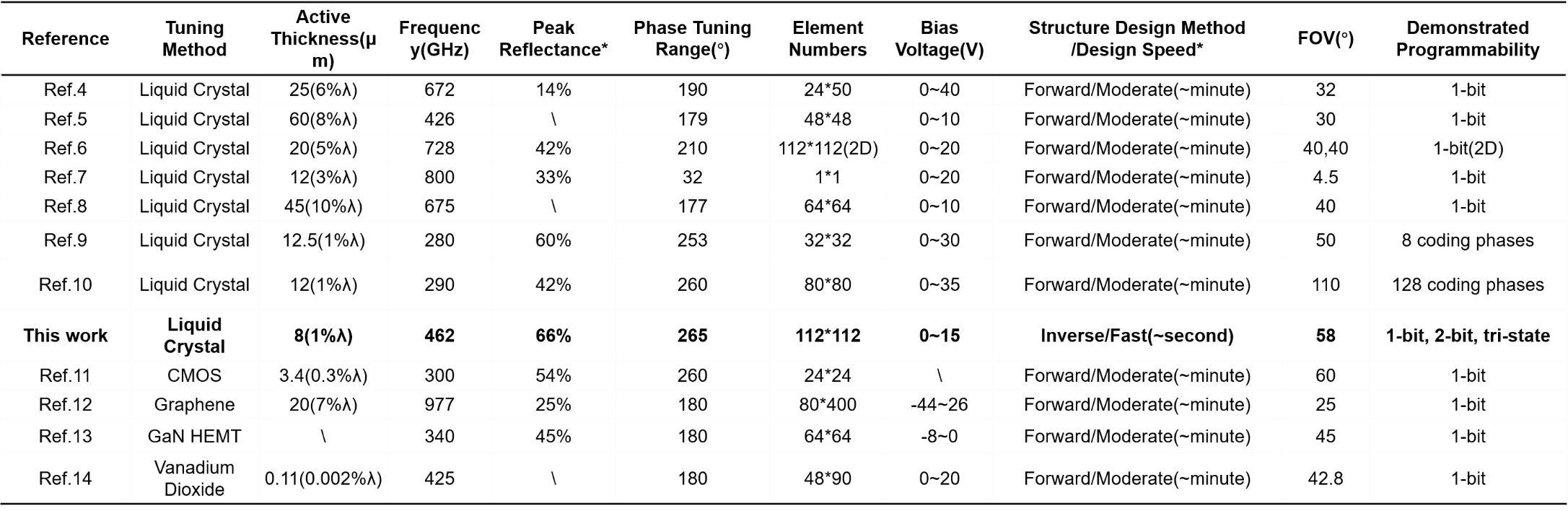


Table S3: Performance comparison of various devices in Terahertz beamsteering

*Value estimated from published results.

References

[1] Haji, S. H., & Abdulazeez, A. M. (2021). Comparison of optimization techniques based on gradient descent algorithm: A review. *PalArch's Journal of Archaeology of Egypt/Egyptology*, 18(4), 2715-2743.

[2] Deng, Y., Ren, S., Fan, K., Malof, J. M., & Padilla, W. J. (2021). Neural-adjoint method for the inverse design of all-dielectric metasurfaces. *Optics Express*, 29(5), 7526-7534.

[3] Park, J., Kang, J. H., Kim, S. J., Liu, X., & Brongersma, M. L. (2017). Dynamic reflection phase and polarization control in metasurfaces. *Nano letters*, 17(1), 407-413.

[4] Wu, J., Shen, Z., Ge, S., Chen, B., Shen, Z., Wang, T., ... & Wu, P. (2020). Liquid crystal programmable metasurface for terahertz beam steering. *Applied physics letters*, 116(13).

[5] Liu, C. X., Yang, F., Fu, X. J., Wu, J. W., Zhang, L., Yang, J., & Cui, T. J. (2021). Programmable manipulations of terahertz beams by transmissive digital coding metasurfaces based on liquid crystals. *Advanced Optical Materials*, 9(22), 2100932.

[6] Li, W., Chen, B., Hu, X., Guo, H., Wang, S., Wu, J., ... & Wu, P. (2023). Modulo-addition operation enables terahertz programmable metasurface for high-resolution two-dimensional beam steering. *Science Advances*, 9(42), eadi7565.

[7] Buchnev, O., Podoliak, N., Kaltenecker, K., Walther, M., & Fedotov, V. A. (2020). Metasurface-based optical liquid crystal cell as an ultrathin spatial phase modulator for THz applications. *ACS Photonics*, *7*(11), 3199-3206.

[8] Fu, X., Shi, L., Yang, J., Fu, Y., Liu, C., Wu, J. W., ... & Cui, T. J. (2022). Flexible terahertz beam manipulations based on liquid-crystal-integrated programmable metasurfaces. *ACS Applied Materials & Interfaces*, 14(19), 22287-22294.

[9] Shen, Z., Li, W., Jin, B., & Zhao, D. (2024). A liquid crystal-based multi-bit terahertz reconfigurable intelligent surface. *APL Photonics*, 9(1).

[10] Chen, C., Chen, S., Ni, Y., Xu, Y., & Yang, Y. (2024). Liquid Crystal Metasurface for On‐Demand Terahertz Beam Forming Over 110° Field‐Of‐View. *Laser & Photonics Reviews*, 2400237.

[11] Venkatesh, S., Lu, X., Saeidi, H., & Sengupta, K. (2020). A high-speed programmable and scalable terahertz holographic metasurface based on tiled CMOS chips. *Nature electronics*, 3(12), 785-793.

[12] Tamagnone, M., Capdevila, S., Lombardo, A., Wu, J., Centeno, A., Zurutuza, A., ... & Mosig, J. R. (2018). Graphene reflectarray metasurface for terahertz beam steering and phase modulation. *arXiv preprint* arXiv:1806.02202.

[13] Lan, F., Wang, L., Zeng, H., Liang, S., Song, T., Liu, W., ... & Mittleman, D. M. (2023). Real-time programmable metasurface for terahertz multifunctional wave front engineering. *Light: Science & Applications*, 12(1), 191.

[14] Chen, B., Wang, X., Li, W., Li, C., Wang, Z., Guo, H., ... & Wu, P. (2022). Electrically addressable integrated intelligent terahertz metasurface. *Science Advances*, 8(41), eadd1296.
